# Needle-tip insertion method for EUS–guided hepaticogastrostomy in puncturing a nondilated bile duct (with video)

**DOI:** 10.1097/eus.0000000000000194

**Published:** 2026-05-14

**Authors:** Hiroaki Tsuji, Ryota Sagami, Takao Sato, Hidefumi Nishikiori, Yasuhisa Hiroshima, Kazuhiro Mizukami

**Affiliations:** 1 Department of Gastroenterology, Oita San-ai Medical Center, Oita, Japan; 2 Department of Advanced Gastrointestinal Cancer Medicine, Faculty of Medicine, Oita University, Oita, Japan; 3 Department of Gastroenterology, Faculty of Medicine, Oita University, Oita, Japan.

A 72-year-old man was admitted with recurrent cholangitis following pancreaticoduodenectomy with hepaticojejunostomy. Magnetic resonance cholangiopancreatography revealed a common bile duct stone (CBDS) measuring 18 mm in diameter [Figure 1A]. During balloon enteroscopy–assisted endoscopic retrograde cholangiography, the guidewire could not be advanced across the CBDS.

**Figure 1.**
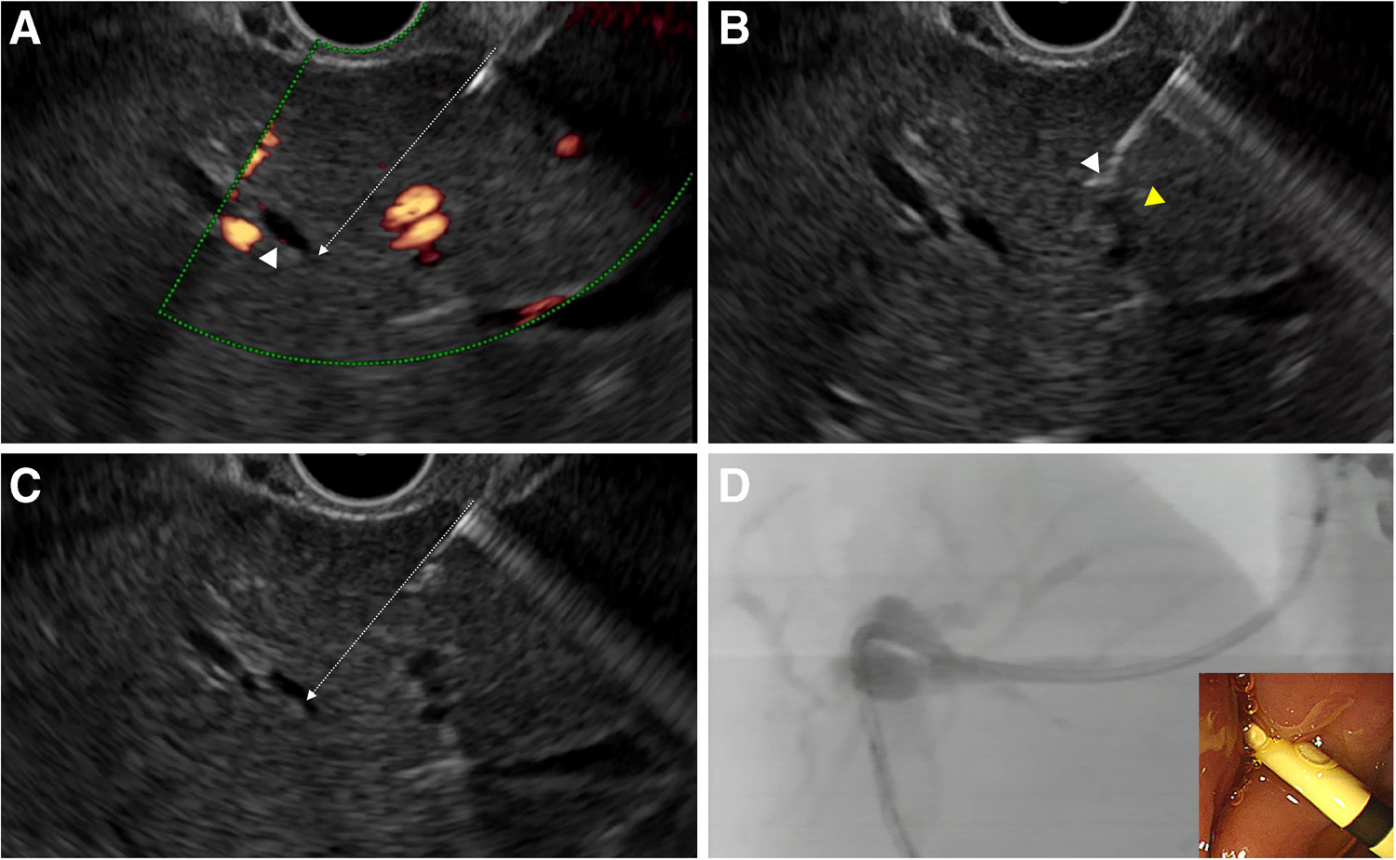
EUS–guided hepaticogastrostomy (EUS-HGS) using the needle-tip insertion method following failed BE-ERCP. A, The needle tip (white arrowhead), positioned close to the gastrointestinal wall and external sheath, allows for a stable puncture view unaffected by respiratory motion and enables precise measurement of the distance and angle to the nondilated target bile duct (dotted white arrow). B, The needle is advanced under continuous visualization (white arrowhead), allowing confirmation and avoidance of small vessels (yellow arrowhead). C, Minor withdrawal of the needle (dotted white arrow) enables fine adjustment of the puncture trajectory for safe entry. D, Following insertion of a double-lumen catheter and an additional guidewire, a dedicated 7-Fr, 14-cm plastic stent was successfully deployed without mechanical or balloon dilation. BE-ERCP, balloon enteroscopy–assisted endoscopic retrograde cholangiography.

EUS–guided hepaticogastrostomy is an effective approach when endoscopic retrograde cholangiography fails.^[1–3]^ The target intrahepatic bile duct was extremely thin, measuring less than 2 mm in diameter. A puncture using the needle-tip insertion method^[1]^ was attempted with a 22-gauge needle (SonoTip; Medico’s Hirata Inc., Osaka, Japan) and a 0.018-inch guidewire (Fielder18; Asahi Intecc Co., Ltd., Aichi, Japan). The needle tip is inserted at a short distance, crimped to the gastrointestinal membrane, which secures a stable puncture view unaffected by respiratory fluctuations and accurate measurement of the distance and angle to the target bile duct [Figure 1A and Video 1]. The needle was advanced under continuous tip visualization, enabling avoidance of vessels and adjustment of the puncture route, even in cases of misalignment between the endoscope and the target axis [Figure 1B, C and Video 1].

**Video 1.** 

After a double-lumen catheter and a second guidewire were inserted, a dedicated 7-Fr, 14-cm plastic stent for EUS–guided hepaticogastrostomy (IT stent; Gadelius Medical, Tokyo, Japan) was placed. Four weeks later, a guidewire was inserted alongside the stent, which was subsequently removed, and EUS-created route was dilated using an 8-mm balloon catheter (REN; Kaneka Medix, Tokyo, Japan). A peroral cholangioscope (SpyGlass DS-II; Boston Scientific, Tokyo, Japan) was inserted without resistance, and the CBDS was fragmented [Figure 2 and Video 1], achieving complete stone clearance. The patient remained asymptomatic without adverse events/recurrence.

**Figure 2.**
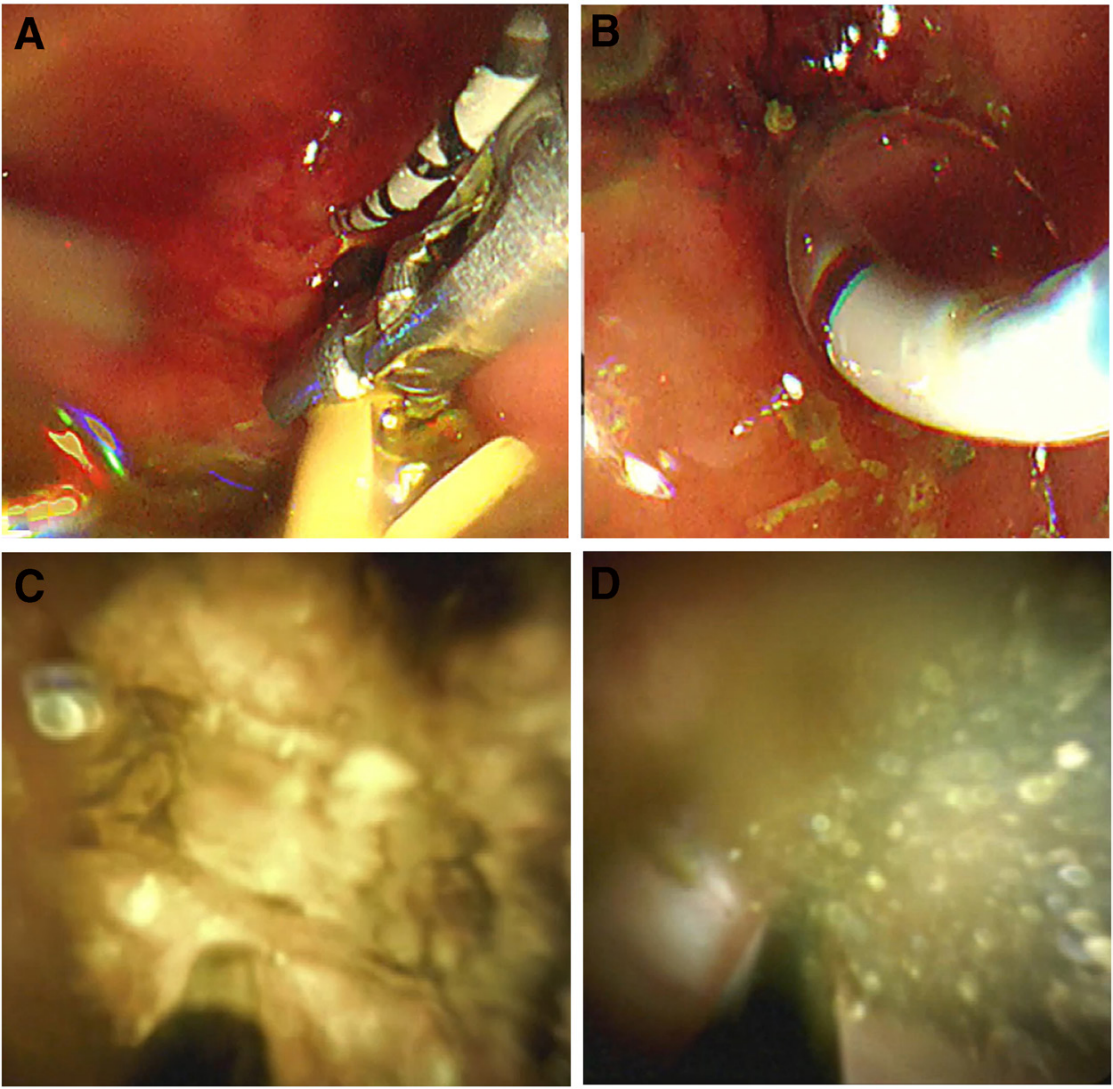
Stone removal *via* EUS-created route (ESCR) using peroral cholangioscopy and electrohydraulic lithotripsy (EHL). A, A guidewire was inserted alongside the indwelling stent, which was then removed. B, ESCR was dilated using an 8-mm balloon catheter. C, D, The large bile duct stone was fragmented using peroral cholangioscopy–guided EHL.

The needle-tip insertion method may also be applicable in cases where balloon enteroscopy–assisted endoscopic retrograde cholangiography fails, especially in nondilated intrahepatic bile ducts.

## Supplementary Videos

Video 1. Demonstration of EUS–guided hepaticogastrostomy with needle-tip insertion method targeting a nondilated intrahepatic bile duct, followed by stone fragmentation using peroral cholangioscopy–guided electrohydraulic lithotripsy *via* EUS-created route. Videos are only available at the official website of the journal (www.eusjournal.com).

## Ethical Statements

This report was approved by the institutional review board and the authors obtained his consent for publication in the journal.

## Conflicts of Interest

The authors declare that they have no conflict of interest with regard to the content of this report.

## Author Contributions

H. Tsuji: writing-original draft and critical revision of the paper. R. Sagami: writing review and editing. T. Sato, H. Nishikiori, Y. Hiroshima, and K. Mizukami participated in study design.

## Data Availability Statement

No datasets were generated or analyzed during this report.
